# Piezoelectric Peptide and Metabolite Materials

**DOI:** 10.34133/2019/9025939

**Published:** 2019-11-21

**Authors:** Hui Yuan, Peipei Han, Kai Tao, Shuhai Liu, Ehud Gazit, Rusen Yang

**Affiliations:** ^1^School of Advanced Materials and Nanotechnology, Xidian University, Xi'an 710126, China; ^2^Department of Molecular Microbiology and Biotechnology, George S. Wise Faculty of Life Sciences, Tel Aviv University, Tel Aviv 6997801, Israel

## Abstract

Piezoelectric materials are important for many physical and electronic devices. Although many piezoelectric ceramics exhibit good piezoelectricity, they often show poor compatibility with biological systems that limits their biomedical applications. Piezoelectric peptide and metabolite materials benefit from their intrinsic biocompatibility, degradability, and convenient biofunctionalization and are promising candidates for biological and medical applications. Herein, we provide an account of the recent progress of research works on piezoelectric peptide and metabolite materials. This review focuses on the growth mechanism of peptide and metabolite micro- and nanomaterials. The influence of self-assembly processes on their piezoelectricity is discussed. Peptide and metabolite materials demonstrate not only outstanding piezoelectric properties but also unique electronic, optical, and physical properties, enabling their applications in nanogenerators, sensors, and optical waveguiding devices.

## 1. Introduction

The discovery of piezoelectricity can be traced back to 1880 by the Curie brothers [1]. They studied the effect of crystal structures on piezoelectric phenomena and further predicted the relation between voltage and stress for piezoelectric materials [2]. Natural biomaterials were found to have polarization in 1941 [3], and shear piezoelectricity was later found by Fukada in various biopolymers like cellulose and collagen in the 1950s [4]. Excellent piezoelectric properties were found in Lead-Zirconate-Titanate (PZT) solid-solution ceramics in 1954 [5], and PZT has since then played an important role in piezoelectric applications [6]. Piezoelectric materials were used in macroscale electromechanical transducers for military and marine applications in early days [7]. Studies and applications of piezoelectric materials were accelerated with the development of microelectromechanical systems (MEMS) [8]. Piezoelectric materials have been widely used in energy harvesters, sensors, transformers, actuators, piezotronics, and so on [7, 9–26]. Piezoelectric ceramics such as barium titanate, PZT, zinc oxide, and molybdenum disulfide have been widely studied. However, their brittleness and nonbiological nature limited their application in biological systems.

Piezoelectricity has been found in biomaterials like virus [27], polyvinylidene fluoride (PVDF) [28, 29], polyhydroxybutyrate (PHB) [30], poly-l-lactic acid (PLLA) [31], peptides [32, 33], amino acid [34], and protein [35]. In addition to piezoelectric properties, some peptide and metabolite materials exhibit excellent conductive, optical, and physical properties, making them excellent candidates for electronic, optical, and other applications. Using amino acids as building blocks, these materials are of good biocompatibility, biodegradability, and chemical transformation. Those properties are highly dependent on their self-assembly processes. Therefore, understanding the self-assembly process and their properties is important for the fundamental study and practical applications.

In this review, we provide an overview of piezoelectric biomaterials in Section 2. In Sections 3 and 4, we discuss the self-assembly processes of peptide and metabolite materials, respectively. In Section 5, we discuss in detail their piezoelectric, semiconductive, optical, thermal, and mechanical properties. Their remarkable properties enable their applications in nanogenerators, sensors, cell imaging, and drug releases. At the end of the article, we highlight current challenges and our perspectives of peptide and metabolite materials and discuss their great potentials in emerging fields. It is expected that this review article will inspire new research efforts for the fundamental understanding and wide application of piezoelectric and functional biomaterials.

## 2. Piezoelectric Biomaterials

Piezoelectricity is found in many materials with noncentrosymmetric crystal structures, and piezoelectric charges are generated when a mechanical stress is applied [36, 37]. Many biomaterials such as peptides, polyvinylidene fluoride (PVDF), poly(lactic acid) (PLA), virus, amino acids, and protein can form noncentrosymmetric crystals and exhibit piezoelectricity. These materials can be classified into natural and synthetic biomaterials. Natural biomaterials usually exhibit weak piezoelectric property and uncontrollable shapes [38]. Synthetic biomaterials have attracted more and more research efforts, and synthetic biomaterials with designable structures and better piezoelectric properties have been found. PVDF-based polymers are among the most important piezoelectric biomaterials owing to their outstanding piezoelectricity, simple structure, and flexibility [39]. It has been found that PVDF and its copolymers have great potentials in promoting cell differentiation, bone growth, and neural and muscle regeneration for tissue engineering and in energy harvesting systems [33, 40–42]. Their crystal structures, synthesized methods, piezoelectric properties, and applications were covered in many good review articles [43–47]. In this review, we mainly focus on piezoelectric peptides and metabolite biomaterials.

Piezoelectric peptides received the most attention owing to their strong piezoelectricity and various nanostructures. They self-assemble due to noncovalent interactions like hydrogen bonding interactions, van der Waals interactions, electrostatic interactions, and hydrophobic and *π*–*π* stacking interactions [48, 49]. Some self-assembled nanostructures have noncentrosymmetric crystal structures and display excellent piezoelectric properties. Those piezoelectric and biocompatible peptides play an important role in fabricating power generators, ultrasensitive sensors, medical delivery systems, cell culture, metal organic frameworks, and energy storage devices. Compared to peptides, crystals based on proteins and amino acids usually displayed weaker piezoelectricity [50, 51]. However, a recent work revealed that a *β*-glycine crystal exhibited a high piezoelectric constant d16 up to 178 pm V^−1^, making it a promising candidate for electronics in bioapplications [52].

## 3. Synthesis of Piezoelectric Peptide Materials

Piezoelectricity has been found in peptides, including diphenylalanine (FF) [53], *cyclo*-glycine-tryptophan (*cyclo*-GW) [54], *β* glycine [52], gamma (*γ*) glycine [55], Fmoc-FF [56], *cyclo*-phenylalanine-tryptophan (FW) [54], and bis-cyclic-*β*-peptides [57]. Among them, FF peptides were the most studied piezoelectric biomaterials.

**Figure 1:**
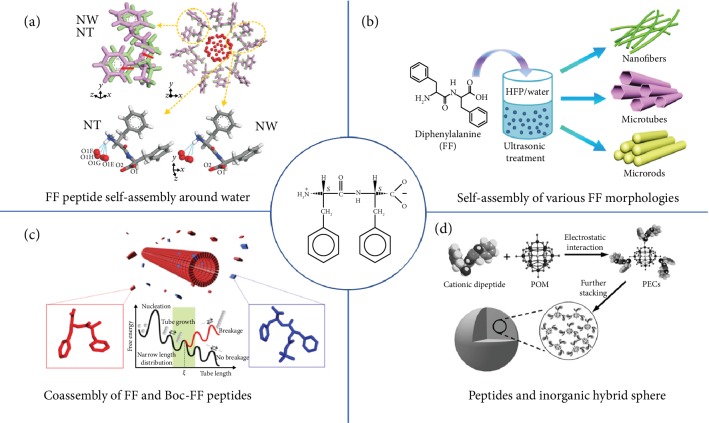
(a) The molecular arrangements of diphenylalanine hosting water molecules in nanotubes and nanowires in the hexagonal lattice system after Rietveld refinement [59]. (b) Schematic diagram of self-assembled FF in nanofibers, microtubes, and microrods [65]. (c) Schematic diagram of coassembled FF and Boc-FF peptide nanotubes. The red molecule represents an FF building block, and the blue molecule represents a Boc-FF building block [74]. (d) Schematic diagram of a cationic dipeptide and polyoxometalates (POMs) coassembling to hybrid supramolecular structures [77].

Since the discovery of FF nanotubes by Reches and Gazit in 2003 through a self-assembly process in solution, the self-assembly process of FF and FF-based nanostructures have attracted significant interests from researchers [58]. The FF nanostructure can be self-assembled into a columnar phase parallel to the long axis of the structure (Figure 1(a)) [59]. During the self-assembly process, FF units stacked along cyclic hexamer structures and hosted H_2_O molecules by strong hydrogen bonds between FF and H_2_O. FF molecules with amine and carboxyl groups form hydrophilic tunnels with H_2_O molecules in them [60, 61]. During the self-assembly process, water content in hydrophilic tunnels affected the morphological diversity and FF-based peptides including fibrils, nanowires, nanotubes, nano/microrods, hollow tubes, quantum dots, and hydrogel have been found [62]. The various morphologies of FF-based peptides were tuned by temperature, PH value, solvent, sonication time, and peptide concentration in solution [63, 64]. FF tubes, wires, and fibers were easily obtained in water, organic solvent like 1,1,1,3,3,3-hexafluoro-2-propanol (HFP), methanol, acetonitrile, and chloroform or mixed solution [65–67] (Figure 1(b)). High concentration, long-time ultrasonication, and suitable HPF/water ratio contributed to the formation of the FF microtube [65]. FF-based nanospheres [68, 69] or quantum dots [70] were self-assembled in solution at low temperature. FF supermolecules self-assemble into a noncentrosymmetric hexagonal (*P*6_1_) structure in solution at low temperature, and good piezoelectric properties were found in many hexagonal FF nanostructures [71, 72]. When the temperature increases over 142°C, the hexagonal FF transfered into orthorhombic (*P*2_1_2_1_2) *cyclo*-FF crystalline structures [73].

Supramolecular polymer coassembly is an efficient approach to control the structure of FF peptides [74, 75]. The coassembly with different molar ratios of N-(*tert*-butoxycarbonyl)-L-Phe-L-Phe-COOH (Boc-FF) and FF led to FF nanotubes with different lengths (Figure 1(c)) [74]. Compared to single FF and Boc-FF tubes, the mixture of FF and Boc-FF tubes tended to form in double-distilled water at 80°C. The phenomenon was caused by the hydrophobic nature. Different peptides formed copolymer nanostructures through self-assembly processes [74]. With the help of *π* − *π* stacking and electrostatic interactions, FF and porphyrin-based porous microspheres were fabricated via a hierarchical coassembly method [76]. Using the electrostatic interaction, a peptide-inorganic sphere was achieved by coassembling cationic FF and polyanionic phosphotungstic acid in water (Figure 1(d)) [77].

**Figure 2:**
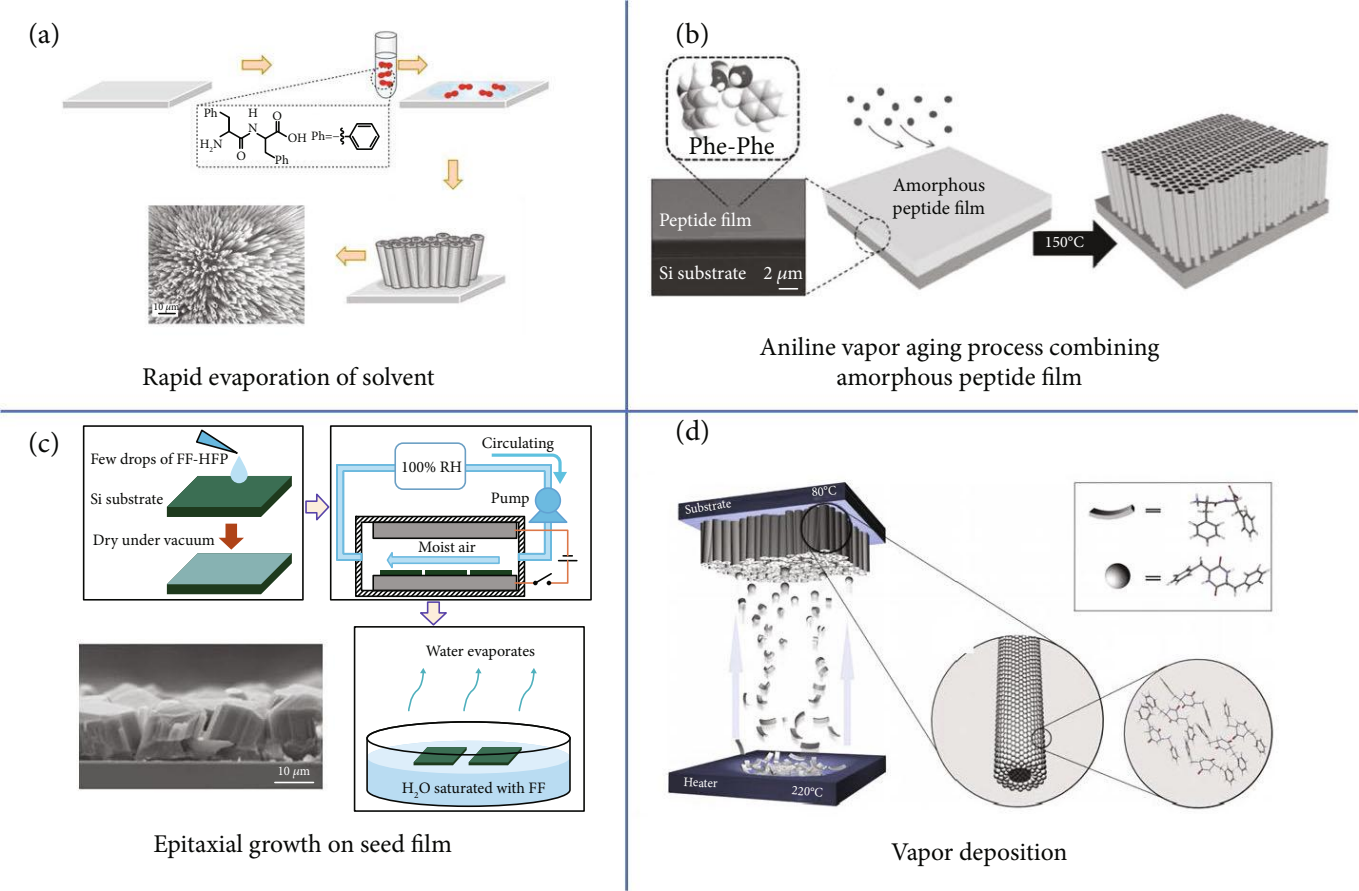
(a) A possible model for the formation of an aligned peptide nanotube array by rapid evaporation of the HFP solvent [78]. (b) Schematic diagram for the growth of vertically well-aligned FF nanowires from an amorphous peptide film by high-temperature aniline vapor aging [80]. (c) Schematic illustration for the fabrication process of a vertical FF peptide array by low-temperature epitaxial growth [72]. (d) Schematic diagram of the vapor deposition technique [81].

Ordered horizontal or vertical FF arrays can grow on substrates. Rapid evaporation of FF solution in 1,1,1,3,3,3-hexafluoro-2-propanol (HFP) solvent led to the growth of vertical peptide nanotube arrays on a siliconized glass substrate (Figure 2(a)) [78]. In this process, the evaporation of the HFP solvent allowed the generation of numerous nucleation sites. FF molecules stacked on the nucleation sites resulted in the self-assembly of vertical FF tubes. The FF peptide can also be fabricated into horizontal FF nanotube arrays. Coating magnetic particles on an FF nanotube with the help of noncovalent interactions resulted in the growth of ordered FF nanotube arrays under external low magnetic fields [78]. By applying high electric fields, *B* > 7 T, the FF building blocks self-assembled into horizontally ordered tubes without coating with magnetic materials [79]. The primary reason can be ascribed to the diamagnetic anisotropy of the aromatic ring [79]. Vertical FF arrays were obtained by growing on an amorphous peptide film [80]. For example, Ryu and Park reported well-aligned nanowire arrays with an orthorhombic phase by exposing an amorphous peptide film to aniline vapor at a high temperature (Figure 2(b)) [80]. The morphology of the FF array was finely controlled by aging temperature and the solvent vapor. When the amorphous peptide film was converted into a crystalline seed film under a humid environment, the FF peptides were grown into vertically aligned microrods on various substrates by low-temperature epitaxial growth (Figure 2(c)) [72]. Vapor deposition was another method to grow vertical peptide arrays [81, 82]. Gazit et al. demonstrated a new vapor deposition method to achieve orthorhombic (*P*2_1_2_1_2) *cyclo*-FF peptides with various lengths and densities through adjusting deposition time (Figure 2(d)) [81]. By combining vapor deposition technology with photolithography, vertically aligned peptide arrays with desirable patterns were obtained [81, 83]. When plasma was applied during the vapor deposition, the nanotube structure of peptides converted into a nanoribbon-like structure with the increase of plasma's frequency [84].

## 4. Synthesis of Piezoelectric Metabolite Materials

There were a number of reports on artificial proteins with piezoelectricity [85]. Silk fibroin, one kind of fibrous protein, has been fabricated into nanostructures that exhibited piezoelectricity, biocompatibility, and degradation in vivo [50, 85]. Piezoelectric properties have been found in artificial amino acids [55]. Glycine, the simplest amino acid, can form crystals with *α*, *β*, and *γ* phases. Among them, both the *β* phase with the noncentral symmetric space group *P*2_1_ and the *γ* phase with the noncentrosymmetric space group *P*3_2_ exhibited piezoelectricity [52]. The single-layer *β*-glycine is metastable and converts easily into *α*-glycine in air [86]. *γ*-Glycine crystals with a trigonal hemihedral symmetry are stable at room temperature, but *γ*-crystals are hard to grow [87]. It was reported that the stable *γ*-glycine crystal was synthesized by either a slow evaporation or a slow cooling progress [55, 88, 89]. The various *γ*-glycine crystals were synthesized by a spin coating technology [90]. Their morphologies were controlled by tuning rotation frequency and changing the wettability of the substrates. Dendritic amino acid films were obtained at low rotation frequency, while ordered micro- and nanoisland arrays were achieved at high rotation frequency. When a mica with a wettable surface was used as a substrate, a 15 nm film was formed [90].

## 5. Properties and Applications of Peptide and Metabolite Materials

### 5.1. Piezoelectricity

**Figure 3:**
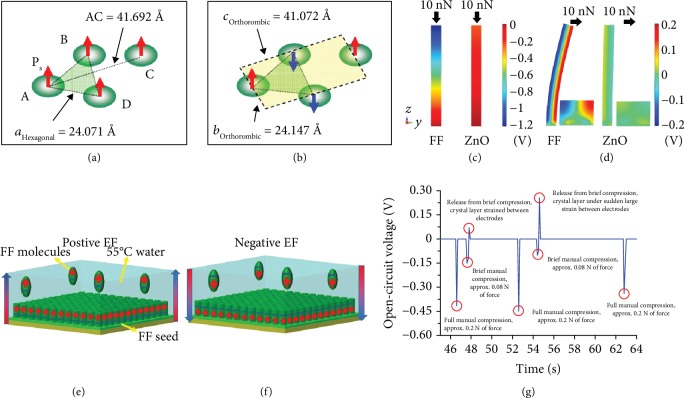
Dipole moment distribution models of the hexagonal (a) and orthorhombic (b) structures of FF peptide nanotubes [91]. (c and d) Simulation of electric potential in nanowires 500 nm long and 50 nm in diameter. (c) Piezoelectric potential in a *y*-*z* plane slice for FF peptide (left) and ZnO (right) nanowires under 10 nN of compressive force. (d) Piezoelectric potential in a *y*-*z* plane slice for FF peptide (left) and ZnO (right) nanowires under 10 nN transverse force. Insets show regions of potential reversal at the nanowire roots [95]. Growth of vertical FF peptide microrod arrays with polarization controlled by the positive EF (electric field) (e) and the negative EF (f). The large arrows are directions of applied electric fields, and the plus and minus signs indicate the polarizations of the FF molecules and FF microrods [53]. (g) Open-circuit voltage response of a layer of *γ*-glycine seed crystals, orientated along the crystallographic b axis. Manual force was applied along the crystallographic b axis over time [52].

It was reported that the piezoelectric constant *d*_33_ of FF peptides ranged from 5 to 30 pm V^−1^ [53, 91, 92]. As-grown FF peptides exhibited often random polarization directions. Applying electric fields during the self-assembly process resulted in the aligned growth of peptide microrods with a uniform polarization, and an effective piezoelectric coefficient *d*_33_ = 17.9 pm V^−1^ was obtained [53]. Kholkin et al. synthesized in a solution FF peptide nanotubes with an effective piezoelectric constant of at least 60 pm V^−1^ [71]. The piezoelectric constant matrix of FF peptides was investigated by Vasilev et al. [93]. They found that the piezoelectric constant *d*_15_ of FF reached 80 ± 15 pm V^−1^, higher than the piezoelectric constant *d*_31_ (4 ± 1 pm V^−1^), *d*_33_ (18 ± 5 pm V^−1^), and *d*_14_ (10 ± 1 pm V^−1^) [93]. The strong piezoelectricity in FF hexagonal structures is ascribed to the strong dipole moments *P*_*s*_ of 6FF rings that point at the same orientation [91]. The orthorhombic structure has antiparallel orientations of *P*_*s*_ in 6FF rings and a zero total polarization (Figures 3(a) and 3(b)) [91]. Piezoelectric constants of FF peptides decreased with the increase of temperature [91]. When the temperature was higher than 140°C, ferroelectric-like behavior in FF peptides was found, owing to the formation of an orthorhombic crystal structure [91, 94]. Piezoelectricity has also been found in other FF-based peptides. When the FF was modified by adding a fluorenyl-methoxycarbonyl (Fmoc) side group, the resultant Fmoc-FF nanofibril was found to have a shear piezoelectric constant *d*_15_ of 33.7 pm V^−1^ [56].

Finite element analysis demonstrated that a single FF nanowire generated an output voltage of -1.3 V under a compressive load of 10 nN, and the output voltage was 5 times higher than that generated by a ZnO nanowire [95]. The voltage due to a transverse loading in an FF peptide nanowire was over 6 times higher than that in a ZnO nanowire (Figures 3(c) and 3(d)) [95]. The high piezoelectric potential and flexibility make the FF peptide nanowire a promising candidate for nanogenerators. Nguyen et al. reported microrod arrays with uniform polarizations by applying electric fields during the self-assembly growth process (Figures 3(e) and 3(f)) [53]. The FF microrod possessed a piezoelectric constant *d*_33_ as high as 17.9 pm V^−1^. FF microrod arrays were further used to fabricate a nanogenerator that produced an open-circuit voltage of 1.4  V [53]. When the piezoelectric nanogenerator was combined with a triboelectric nanogenerator, a new hybrid nanogenerator was produced and an output voltage up to 2.2 V was obtained [96]. Horizontal FF peptide arrays have also been used in nanogenerators [97]. Horizontal and unidirectionally polarized FF nanotube arrays were reported using a meniscus-driven self-assembly process by Lee et al. [97]. They fabricated peptide fiber arrays into a nanogenerator device that produced a voltage of 2.8 V under a force of 42 N [97]. Other peptides were also recently reported for the fabrication of generators. *Cyclo*-GW, one kind of piezoelectric peptides with a monoclinic (*P*2_1_) crystal structure, has an effective piezoelectric constant of 14.1 pC N^−1^ [54]. When the *cyclo*-GW peptide was fabricated into a nanogenerator, an output voltage of 1.2 V at a force of 65 N was demonstrated [54]. W-based aromatic dipeptides such as *cyclo*-FW peptides with an orthorhombic crystal structure were also used to build an energy harvesting device that produced a high open-circuit voltage reaching 1.4 V [98].

Piezoelectricity has been found in proteins and collagens, and their piezoelectric constants were relatively low in the range of 0.1-12 pm V^−1^ [35, 99, 100]. A collagen fibril from rat tail tendons exhibited a maximum piezoelectric constant of 12 pm V^−1^ [101]. A silk film fabricated by a two-step method exhibited a dynamic shear piezoelectric constant *d*_14_ ranging from 0.01 to 1.5 pC N^−1^ under the draw ratio *λ* from 1 to 2.7 [85]. The weak piezoelectricity and complicated synthesis process of proteins limited their applications as a piezoelectric material. Compared to proteins, the synthesis of glycine crystals was relatively easy [55]. The piezoelectricity in glycine was found in 1954 for the first time [55]. A previous report showed that *γ*-glycine exhibited a low longitudinal piezocoefficient of 10 pm V^−1^ [55]. Recently, a simple *β*-glycine crystal was synthesized by Guerin et al. [52]. They achieved a high shear piezoelectric constant *d*_16_ of up to 178 pm V^−1^. The transverse shear coefficient *d*_16_ was predicted to be 195 pm V^−1^ and its voltage constant was predicted to be 8.13 V mN^−1^ according to a density functional theory (DFT) computation. However, the *β*-glycine crystal was a metastable polymorph [52]. They also synthesized *γ*-glycine single crystals and obtained a piezoelectric constant *d*_33_ of 9.93 pm V^−1^ [52]. The amino acid-based device enabled a maximum output voltage of ~0.45 V under a force of ~0.172 N (Figure 3(g)) [52].

### 5.2. Semiconductivity

**Figure 4:**
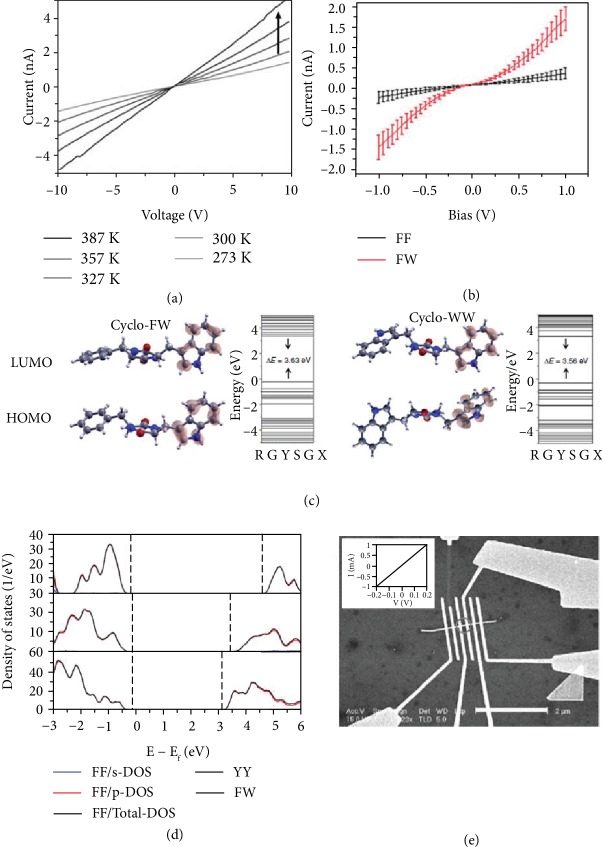
(a) *I* − *V* curves of an orthorhombic peptide NW at various temperatures [102]. (b) The averaged *I* − *V* of FF and FW peptide nanodots [103]. (c) Calculated molecular orbital amplitude plots and energy levels of the highest occupied and lowest unoccupied molecular orbitals of *cyclo*-FW and *cyclo*-WW, showing band gaps of 3.63 eV and 3.56 eV, respectively [105]. (d) Projected DOS for FF (top), YY (middle), and FW (bottom) nanotubes [106]. (e) Scanning electron microscopy image of the actual device (scale bar: 2 *μ*m). (Inset) Current-voltage curve of the silver-seeded silver 4 × 4 nanoribbon [115].

In addition to piezoelectric properties, semiconductivity was also found in peptides. A *cyclo*-FF nanowire obtained by annealing linear FF powders at high temperature displayed semiconductive properties [102]. The current-voltage (*I* − *V*) curve of FF peptides showed that the current increased from -1.5 nA to -5.0 nA at a constant voltage of 10 V when the temperature increases from 273 K to 387 K (Figure 4(a)) [102]. Compared to FF, the conductance of FW was nearly three times higher than that of FF (0.5 nS) and reached 1.4 nS (Figure 4(b)) [103].

The semiconductivity of peptides was evaluated by DFT calculations. *Cyclo*-FF possessed a wide bandgap of 6.41 eV [104], while *cyclo*-FW exhibited a small bandgap of 3.63 eV [105]. The calculated bandgap of *cyclo*-WW (3.56 eV) was narrower than that of *cyclo*-FW (3.63 eV) (Figure 4(c)) [105]. The lower bandgap of *cyclo*-WW was due to the increased hydrogen bonding and aromatic interactions in *cyclo*-WW, when F was replaced by W [98]. In contrast to the *cyclo*-structure, the linear structure of peptides exhibited a smaller bandgap, owing to the easy electron transport in the linear structure and easy hole transport in the *cyclo*-structure [104]. In a calculated linear peptide mode, FW tubes showed the lowest energy bandgap (3.04 eV), followed by dityrosine (YY) and FF tubes (Figure 4(d)) [106]. It was noted that YY had an energy bandgap of 3.24 eV, slightly higher than the energy bandgap of FW. In addition, the energy bandgap of YY was lower than that of FF (4.48 eV) [106]. Those peptides belong to wide-gap biomaterials [61, 107]. Researchers have been devoted in studying peptide's electronic properties [108]. Researches showed that the existence of water molecules in central hydrophilic channels decreased the band gap of FF and promoted the probability of electron hopping, leading to the increase of conductivity [109]. The conductivity of orthorhombic FF peptides increased by doping with poly(allylamine hydrochloride) (PAH) agents [110]. A significant decrease in the peptide's bandgap was achieved, and the decreased value reached 1 eV.

Semiconductive properties have been studied not only in piezoelectric peptides, but also in piezoelectric amino acids and proteins. The bandgap for a *γ*-glycine crystal was calculated as 5.02 eV, indicating insulation characteristics [111]. However, some previous studies revealed that proteins possessed inherent conductivity properties [112–114]. Protein arrays of DNA templating can self-assemble into 4 × 4 ribbons that serve as templates for a highly conductive single silver nanoribbon (Figure 4(e)) [115]. This device was measured with a voltage ranging from -0.2 to 0.2 V and the resistance was found to be 200 Ohm, corresponding to a resistivity of 2.4 × 10^−6^ Ohm · m for silver nanowires.

### 5.3. Optical Properties

**Figure 5:**
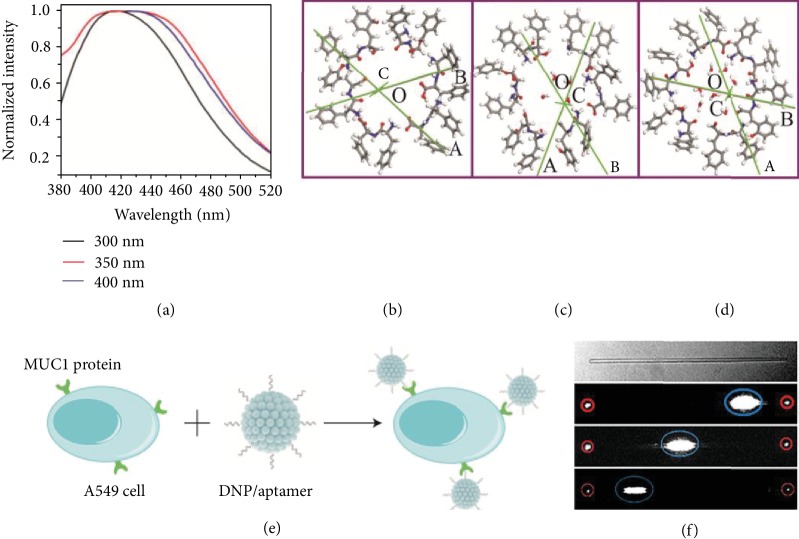
(a) Fluorescent emission spectra of *cyclo*-GW peptides at different excitations [54]. (b–d) Supercells consisting of six FF molecules with (b) 0, (c) 4, and (d) 8 water molecules at the channel core of a supercell [119]. (e) DNPs modified with the MUC1 aptamer (wavy lines) bind to the MUC1 protein (green structure) found on the cell membrane of A549 cells (light blue, cytoplasm; dark blue, cell nucleus) [120]. (f) Optical waveguiding of *cyclo*-FW crystals [98].

Self-assembly piezoelectric peptides showed attractive optical properties, like photoluminescent (PL) and optical waveguiding, owing to the inherent hydrogen bonding and aromatic supramolecular packing networks [108]. After packing, the energy losses caused by intermolecular energy transfer was impeded by the limitation of molecular rotations and vibrations, leading to the excitation of photons [54]. The photon excitation with different wavelength ranges for peptides was found. A peak of excitation wavelength located at 284 nm was found in FF monomers that were excited at 260 nm [116]. While monomers were self-assembled into aligned nanotubes, two red shifted peaks containing a main peak at 305 nm located at the ultraviolet (UV) region and a second peak at 400-500 nm located at the blue region were observed [116]. When linear FF powders grew into *cyclo*-FF nanotubes with a vapor-transported process, a strong blue emission at 465 nm was found [102]. Emission in blue light region (420 nm) was also found in *cyclo*-GW when it was excited at 300-400 nm (Figure 5(a)) [54]. In addition to UV and blue emission, red emission was found under excitation at 515-560 nm in an FF nanotube, because of its inherent guest dye fluorescence [117].

The PL intensity and peak position of self-assembled peptides were adjusted by introducing dopants, modifying the aromatic moieties, or controlling the contents of water [105, 118]. The UV emission peak position of an FF peptide nanotube was influenced by the presence of H_2_O molecules in their channel core (Figures 5(b)–5(d)) [119]. With the increase of H_2_O molecule contents, the UV PL peak position tended to redshift owing to the splitting of valence-band peaks with the increase of H_2_O molecules. When FW peptide nanoparticles were self-assembled with Zn(II), a blue fluorescence emission was found at 370 nm [120]. A high fluorescence intensity in FW peptide nanoparticles modified with MUC1 aptamers enabled their use in fluorescence bioimaging for recognizing and sensing cancer cells (Figure 5(e)). Besides, the FW nanoparticles conjugated with the anthracycline chemotherapy drug DOX enabled the real-time monitoring of drug release [120].

In addition to PL properties, self-assembly peptides exhibited optical waveguiding properties. An optical waveguiding phenomenon was found in *cyclo*-FW peptides when the peptide was excited with a laser. The position and intensity of laser excitation on samples affected the intensity of the excitation laser output at both ends of *cyclo*-FW (Figure 5(f)) [98]. By adding formaldehyde into an FF organogel, FF peptide platelets with thicknesses from tens to hundreds of nanometers were obtained [121]. Thin FF peptides have optical waveguiding properties, enabling red emission and light propagation to the end of nanobelts [121]. The intensity of optical waveguiding was influenced by the incident angle. With the incident angle increased, more output light transmitted to its end sides [122]. When FF was doped with rhodamine B (RhB), FF-RhB microrods enabled an optical waveguide property at the excited wavenumber of 561 nm [65].

### 5.4. Physical Properties

Thermostability and mechanical stability are important for practical applications of materials in the field of flexible devices, especially in nanogenerators and strain sensors. *γ*-Glycine crystals synthesized by a gel method showed a thermal stability of up to 170°C [87]. Peptide nanotubes enabled stabilization in diverse organic solvents and at high temperatures of up to 300°C [123]. The morphology of FF nanotubes was kept at a temperature up to 150°C [67]. When the temperature was higher than 150°C, the crystal structure of FF transformed from a hexagonal structure to an orthorhombic structure, indicating the limited thermal stability of linear FF peptides [124]. Compared to linear FF, *cyclo*-peptides like *cyclo*-GW and *cyclo*-FW peptides have better thermal stability, and they can bear a temperature up to 370°C [54, 98]. Like linear FF, the *γ*-glycine crystal was transformed into *α*-form as the temperature was higher than ~168°C [125].

Peptides and metabolite materials exhibited fascinating mechanical properties. A Young's modulus of ~19 GPa was found in FF peptide tubes [126]. FF nanotubes synthesized in solution exhibited a Young's modulus of 27 GPa and a shear modulus of 0.21 GPa [127]. The unique stiffness and robustness of FF were investigated by DFT calculations. The reason was ascribed to an array of rigid nanotube backbones with an interpenetrating “zipper-like” aromatic interlock in FF nanotubes [128]. Besides, the stiffness is highly dependent on the existence of hydrogen bonds in molecular structures, and the maximal Young's modulus was achieved when a stress was applied along the hydrogen bonding network [129]. The improvement in the Young's modulus was achieved by forming networks of hydrogen bonds [130]. Boc-Phe-Phe-OH (Boc-FF) nanospheres with lateral hydrogen bonding networks and a parallel orientation of building blocks exhibited a Young's modulus as high as 275 GPa, making it a fascinatingly stiff and strong organic material [69]. FF peptides with high mechanical tolerance allowed them to be used as nanofillers to improve the mechanical properties of epoxy [131]. Both the shear and peel strength were increased by 70% and 450%, respectively, as compared to undecorated epoxy [131]. The Young's modulus of amino acid crystals was found to be unusually large and highly anisotropic [129]. *α*-Glycine exhibited a high Young's modulus of 44 GPa along the (001) face and 29 GPa along the (010) face. The *γ*-glycine showed a Young's modulus of 28 GPa along the (100) face [129]. Based on the calculation, the minimum Young's modulus is in the order of 10-20 GPa and the maximum Young's modulus is in the order of 70-90 GPa. The phenomenon of mechanical anisotropy was found in collagen fibrils [132]. They showed a calculated shear modulus of up to 33 MPa and an experimental shear modulus of 2.9 MPa in phosphate-buffered saline, 3.4 MPa in phosphate-buffered saline, and 74 MPa at ambient conditions, respectively [132]. Crystal structures and properties of peptides and metabolite materials are summarized in Table 1.

## 6. Conclusion and Outlook

The inherent piezoelectricity found in peptide and metabolite materials enables their applications in fields of nanogenerators and sensors. Their piezoelectric properties were controlled by their chemical composition, crystal structure, and growing process. Taking advantage of their intrinsic biocompatibility and degradability, these biomaterials are promising candidates for implantable devices for human health monitoring and tissue engineering. Degradable sensors based on piezoelectric biomaterials may precisely monitor tissue regeneration status in real time and decompose into harmless amino acids at the end of its life. However, several challenges remain for the application of peptide and metabolite materials. First, the mechanism of piezoelectricity in biomaterials needs to be further explored. Biomaterials are distinct from piezoelectric ceramics, and more and more piezoelectric biomaterials are discovered in recent years. Molecular dynamics simulation may help reveal the mechanism of piezoelectricity in biomaterials. Second, large-scale and ordered biomaterial arrays with a uniform polarization and strong piezoelectricity need to be developed. High-performance sensors and nanogenerators ask for materials of good piezoelectricity and uniform polarization. Well-designed piezoelectric biomaterials with desired features are still difficult to achieve. Electrical and magnetic fields were proven to affect the polarization of biomaterials and the alignment of nanostructures, and new growth methods need to be developed to achieve piezoelectric biomaterials with controlled properties. Third, thermally and chemically stable piezoelectric biomaterials need to be studied in order to put them into practical applications. Biomaterials, especially amino acids, with stable crystal structures and properties in working environments need to be investigated. Last but not the least, unique properties and corresponding applications of peptide and metabolite materials need to be explored. Developing multifunctional devices to meet various application requirements is an important development trend nowadays.

Good piezoelectric properties, conductivity, optical properties, and mechanical properties of peptide and metabolite materials endow them with great potentials for various devices. Combining piezoelectricity with semiconductivity, the peptide and metabolite materials can be used for the fabrication of biomaterial-based piezotronic devices. The peptides with outstanding optical properties allow their application in cell imaging. Piezoelectric peptide and metabolite materials with fascinating electronic, optical, and physical properties are promising for fabricating biocompatible, degradable, and multifunctional devices, such as an implantable device for real-time health monitoring.

## Figures and Tables

**Table 1:** Summary of some peptides and metabolite materials.

Materials	Crystal structures	Properties of interests	Ref.
FF microrods	Hexagonal (*P*6_1_)	Piezoelectricity (*d*_33_ = 17.9 pm V^−1^)	[53]
Fmoc-FF		Piezoelectricity (*d*_15_ = 33.7 ± 0.7 pm V^−1^)	[56]
*cyclo*-GW	Monoclinic (*P*2_1_)	Piezoelectricity (*d*_36_ = 14.1 pC N^−1^)	[54]
*cyclo*-FW crystals	Orthorhombic (*P*2_1_2_1_2_1_)	Piezoelectricity	[98]
*β*-Glycine	Monoclinic (*P*2_1_)	Piezoelectricity (*d*_16_ = 178 pm V^−1^)	[52]
*γ*-Glycine	Trigonal (*P*3_2_)	Piezoelectricity (*d*_33_ = 9.93 pm V^−1^)	[52]
*cyclo*-FF	Orthorhombic (*P*2_1_2_1_2)	Ferroelectricity	[73]
*cyclo*-FF	Orthorhombic (*P*2_1_2_1_2)	Conductivity (Δ*E* = 6.41 eV)	[104]
*cyclo*-FW	Orthorhombic (*P*2_1_2_1_2_1_)	Conductivity (Δ*E* = 3.63 eV)	[105]
FF nanotubes	Hexagonal (*P*6_1_)	Optical properties (ultraviolet and blue emission)	[116]
*cyclo*-FF	Orthorhombic (*P*2_1_2_1_2)	Optical properties (blue emission)	[101]
*cyclo*-GW	Monoclinic (*P*2_1_)	Optical properties (blue fluorescence)	[54]
*cyclo*-FW	Orthorhombic (*P*2_1_2_1_2_1_)	Optical waveguiding properties	[98]
*γ*-Glycine crystals	Trigonal (*P*3_1_)	Thermally stable up to 170°C	[87]
FF nanotubes	Hexagonal (*P*6_1_)	Thermally stable up to 150°C	[67]
*cyclo*-GW	Monoclinic (*P*2_1_)	Thermally stable up to 370°C	[54]
*cyclo*-FW	Orthorhombic (*P*2_1_2_1_2_1_)	Thermally stable up to 370°C	[98]
FF nanotubes	Hexagonal (*P*6_1_)	Young's modulus: 27 GPa	[127]
*γ*-Glycine		Young's modulus along the (100) plane: 28 GPa	[129]

## References

[1] Curie J., Curie P. (1880). Development by pressure of polar electricity in hemihedral crystals with inclined faces. *Bulletin de la Société Minéralogique de France*.

[2] Schulz M. J., Sundaresan M. J., Mcmichael J., Clayton D., Sadler R., Nagel B. (2003). Piezoelectric materials at elevated temperature. *Journal of Intelligent Material Systems and Structures*.

[3] Martin A. (1941). Tribo-electricity in wool and hair. *Proceedings of the Physical Society*.

[4] Fukada E. History and recent progress in piezoelectric polymer research.

[5] Jaffe B., Roth R. S., Marzullo S. (1954). Piezoelectric properties of lead zirconate-lead titanate solid-solution ceramics. *Journal of Applied Physics*.

[6] Nuffer J., Bein T. Application of piezoelectric materials in transportation industry.

[7] Heywang W., Lubitz K., Wersing W. (2008). *Piezoelectricity. Evolution and Future of a Technology, Vol. 114*.

[8] Muralt P. (2008). Recent progress in materials issues for piezoelectric MEMS. *Journal of the American Ceramic Society*.

[9] Wang Z. L., Song J. (2006). Piezoelectric nanogenerators based on zinc oxide nanowire arrays. *Science*.

[10] Yang R., Qin Y., Li C., Zhu G., Wang Z. L. J. N. L. (2009). Converting biomechanical energy into electricity by a muscle-movement-driven nanogenerator. *Nano Letters*.

[11] Zhu G., Yang R., Wang S., Wang Z. L. (2010). Flexible high-output nanogenerator based on lateral ZnO nanowire array. *Nano Letters*.

[12] Nguyen V., Yang R. (2013). Effect of humidity and pressure on the triboelectric nanogenerator. *Nano Energy*.

[13] Yang R., Qin Y., Dai L., Wang Z. L. (2009). Power generation with laterally packaged piezoelectric fine wires. *Nature Nanotechnology*.

[14] Zhang W., Zhu R., Nguyen V., Yang R. (2014). Highly sensitive and flexible strain sensors based on vertical zinc oxide nanowire arrays. *Sensors and Actuators A: Physical*.

[15] Zhou J., Gu Y., Fei P. (2008). Flexible piezotronic strain sensor. *Nano Letters*.

[16] Zhou J., Fei P., Gu Y. (2008). Piezoelectric-potential-controlled polarity-reversible Schottky diodes and switches of ZnO wires. *Nano Letters*.

[17] Yuan H., Lei T., Qin Y., Yang R. (2019). Flexible electronic skins based on piezoelectric nanogenerators and piezotronics. *Nano Energy*.

[18] Zhu R., Yang R. (2018). *Synthesis and Characterization of Piezotronic Materials for Application in Strain/Stress Sensing*.

[19] Jaffe B. (2012). *Piezoelectric Ceramics, Vol. 3*.

[20] Ikeda T., Ikeda T. (1990). *Fundamentals of Piezoelectricity*.

[21] Yang J. (2005). *An Introduction to the Theory of Piezoelectricity, Vol. 9*.

[22] Erturk A., Inman D. J. (2011). *Piezoelectric Energy Harvesting*.

[23] Steinem C., Janshoff A. (2007). *Piezoelectric Sensors*.

[24] Vazquez C. A. (2016). Piezoelectric transformers: an historical review. *Actuators*.

[25] Uchino K. (1996). *Piezoelectric Actuators and Ultrasonic Motors, Vol. 1*.

[26] Wang Z. L. (2012). Basic theory of piezotronics. *Piezotronics and Piezo-Phototronics*.

[27] Lee B. Y., Zhang J., Zueger C. (2012). Virus-based piezoelectric energy generation. *Nature Nanotechnology*.

[28] Huang T., Wang C., Yu H., Wang H., Zhang Q., Zhu M. (2015). Human walking-driven wearable all-fiber triboelectric nanogenerator containing electrospun polyvinylidene fluoride piezoelectric nanofibers. *Nano Energy*.

[29] Ding R., Zhang X., Chen G. (2017). High-performance piezoelectric nanogenerators composed of formamidinium lead halide perovskite nanoparticles and poly(vinylidene fluoride). *Nano Energy*.

[30] Knowles J., Mahmud F., Hastings G. (1991). Piezoelectric characteristics of a polyhydroxybutyrate-based composite. *Clinical Materials*.

[31] Lee S. J., Arun A. P., Kim K. J. (2015). Piezoelectric properties of electrospun poly(l-lactic acid) nanofiber web. *Materials Letters*.

[32] Bystrov V. S., Bdikin I. K., Heredia A. (2012). Piezoelectricity and ferroelectricity in biomaterials: from proteins to self-assembled peptide nanotubes. *Piezoelectric nanomaterials for biomedical applications*.

[33] Yuan H., Lei T., Qin Y., He J.-H., Yang R. (2019). Design and application of piezoelectric biomaterials. *Journal of Physics D: Applied Physics*.

[34] Isakov D., de Matos Gomes E., Bdikin I. (2011). Production of polar *β*-glycine nanofibers with enhanced nonlinear optical and piezoelectric properties. *Crystal Growth & Design*.

[35] Fukada E., Yasuda I. (1964). Piezoelectric effects in collagen. *Japanese Journal of Applied Physics*.

[36] Wang Z. L. (2012). Progress in piezotronics and piezo-phototronics. *Advanced Materials*.

[37] Wu W., Wang Z. L. (2016). Piezotronics and piezo-phototronics for adaptive electronics and optoelectronics. *Nature Reviews Materials*.

[38] Fukada E. (1983). Piezoelectric properties of biological polymers. *Quarterly Reviews of Biophysics*.

[39] Martins P. M., Ribeiro S., Ribeiro C. (2013). Effect of poling state and morphology of piezoelectric poly(vinylidene fluoride) membranes for skeletal muscle tissue engineering. *RSC Advances*.

[40] Ribeiro C., Sencadas V., Correia D. M., Lanceros-Méndez S. (2015). Piezoelectric polymers as biomaterials for tissue engineering applications. *Colloids and Surfaces B: Biointerfaces*.

[41] Laroche G., Marois Y., Guidoin R. (1995). Polyvinylidene fluoride (PVDF) as a biomaterial: from polymeric raw material to monofilament vascular suture. *Journal of Biomedical Materials Research*.

[42] Persano L., Dagdeviren C., Su Y. (2013). High performance piezoelectric devices based on aligned arrays of nanofibers of poly(vinylidenefluoride-co-trifluoroethylene). *Nature Communications*.

[43] Yan J., Liu M., Jeong Y. G. (2019). Performance enhancements in poly(vinylidene fluoride)-based piezoelectric nanogenerators for efficient energy harvesting. *Nano Energy*.

[44] Houis S., Engelhardt E. M., Wurm F., Gries T. (2010). Application of polyvinylidene fluoride (PVDF) as a biomaterial in medical textiles. *Medical and Healthcare Textiles*.

[45] Li Y., Liao C., Tjong S. C. (2019). Electrospun polyvinylidene fluoride-based fibrous scaffolds with piezoelectric characteristics for bone and neural tissue engineering. *Nanomaterials*.

[46] Ruan L., Yao X., Chang Y., Zhou L., Qin G., Zhang X. (2018). Properties and applications of the *β* phase poly(vinylidene fluoride). *Polymers*.

[47] Kochervinskii V. V. (2003). Piezoelectricity in crystallizing ferroelectric polymers: poly(vinylidene fluoride) and its copolymers (a review). *Crystallography Reports*.

[48] Adler-Abramovich L., Gazit E. (2014). The physical properties of supramolecular peptide assemblies: from building block association to technological applications. *Chemical Society Reviews*.

[49] Cavalli S., Albericio F., Kros A. (2010). Amphiphilic peptides and their cross-disciplinary role as building blocks for nanoscience. *Chemical Society Reviews*.

[50] Horan R. L., Antle K., Collette A. L. (2005). In vitro degradation of silk fibroin. *Biomaterials*.

[51] Marino A. A., Becker R. O., Soderholm S. C. (1971). Origin of the piezoelectric effect in bone. *Calcified Tissue Research*.

[52] Guerin S., Stapleton A., Chovan D. (2017). Control of piezoelectricity in amino acids by supramolecular packing. *Nature Materials*.

[53] Nguyen V., Zhu R., Jenkins K., Yang R. (2016). Self-assembly of diphenylalanine peptide with controlled polarization for power generation. *Nature Communications*.

[54] Tao K., Hu W., Xue B. (2019). Bioinspired stable and photoluminescent assemblies for power generation. *Advanced Materials*.

[55] Heredia A., Meunier V., Bdikin I. K. (2012). Nanoscale ferroelectricity in crystalline *γ*-glycine. *Advanced Functional Materials*.

[56] Ryan K., Beirne J., Redmond G. (2015). Nanoscale piezoelectric properties of self-assembled Fmoc-FF peptide fibrous networks. *ACS Applied Materials & Interfaces*.

[57] Tabata Y., Takagaki K., Uji H., Kimura S. (2019). Piezoelectric property of bundled peptide nanotubes stapled by bis-cyclic-*β*-peptide. *Journal of Peptide Science*.

[58] Reches M., Gazit E. (2003). Casting metal nanowires within discrete self-assembled peptide nanotubes. *Science*.

[59] Kim J., Han T. H., Kim Y. I. (2010). Role of water in directing diphenylalanine assembly into nanotubes and nanowires. *Advanced Materials*.

[60] Görbitz C. H. (2001). Nanotube formation by hydrophobic dipeptides. *Chemistry - A European Journal*.

[61] Silva R. F., Araújo D. R., Silva E. R., Ando R. A., Alves W. A. (2013). L-Diphenylalanine microtubes as a potential drug-delivery system: characterization, release kinetics, and cytotoxicity. *Langmuir*.

[62] Souza M. I., Silva E. R., Jaques Y. M., Ferreira F. F., Fileti E. E., Alves W. A. (2014). The role of water and structure on the generation of reactive oxygen species in peptide/hypericin complexes. *Journal of Peptide Science*.

[63] Kumaraswamy P., Lakshmanan R., Sethuraman S., Krishnan U. M. (2011). Self-assembly of peptides: influence of substrate, pH and medium on the formation of supramolecular assemblies. *Soft Matter*.

[64] Wang M., Du L., Wu X., Xiong S., Chu P. K. (2011). Charged diphenylalanine nanotubes and controlled hierarchical self-assembly. *ACS Nano*.

[65] Li Q., Jia Y., Dai L., Yang Y., Li J. (2015). Controlled rod nanostructured assembly of diphenylalanine and their optical waveguide properties. *ACS Nano*.

[66] Yan X., Cui Y., He Q., Wang K., Li J. (2008). Organogels based on self-assembly of diphenylalanine peptide and their application to immobilize quantum dots. *Chemistry of Materials*.

[67] Adler-Abramovich L., Reches M., Sedman V. L., Allen S., Tendler S. J. B., Gazit E. (2006). Thermal and chemical stability of diphenylalanine peptide nanotubes: implications for nanotechnological applications. *Langmuir*.

[68] Reches M., Gazit E. (2004). Formation of closed-cage nanostructures by self-assembly of aromatic dipeptides. *Nano Letters*.

[69] Adler-Abramovich L., Kol N., Yanai I. (2010). Self-assembled organic nanostructures with metallic-like stiffness. *Angewandte Chemie International Edition*.

[70] Amdursky N., Molotskii M., Gazit E., Rosenman G. (2009). Self-assembled bioinspired quantum dots: optical properties. *Applied Physics Letters*.

[71] Kholkin A., Amdursky N., Bdikin I., Gazit E., Rosenman G. (2010). Strong piezoelectricity in bioinspired peptide nanotubes. *ACS Nano*.

[72] Nguyen V., Jenkins K., Yang R. (2015). Epitaxial growth of vertically aligned piezoelectric diphenylalanine peptide microrods with uniform polarization. *Nano Energy*.

[73] Bdikin I., Bystrov V., Kopyl S. (2012). Evidence of ferroelectricity and phase transition in pressed diphenylalanine peptide nanotubes. *Applied Physics Letters*.

[74] Adler-Abramovich L., Marco P., Arnon Z. A. (2016). Controlling the physical dimensions of peptide nanotubes by supramolecular polymer coassembly. *ACS Nano*.

[75] Yuran S., Razvag Y., Reches M. (2012). Coassembly of aromatic dipeptides into biomolecular necklaces. *ACS Nano*.

[76] Zou Q., Zhang L., Yan X. (2014). Multifunctional porous microspheres based on peptide-porphyrin hierarchical co-assembly. *Angewandte Chemie International Edition*.

[77] Yan X., Zhu P., Fei J., Li J. (2010). Self-assembly of peptide-inorganic hybrid spheres for adaptive encapsulation of guests. *Advanced Materials*.

[78] Reches M., Gazit E. (2006). Controlled patterning of aligned self-assembled peptide nanotubes. *Nature Nanotechnology*.

[79] Hill R. . J. A., Sedman V. . L., Allen S. (2007). Alignment of aromatic peptide tubes in strong magnetic fields. *Advanced Materials*.

[80] Ryu J., Park C. B. (2008). High-temperature self-assembly of peptides into vertically well-aligned nanowires by aniline vapor. *Advanced Materials*.

[81] Adler-Abramovich L., Aronov D., Beker P. (2009). Self-assembled arrays of peptide nanotubes by vapour deposition. *Nature Nanotechnology*.

[82] Bank-Srour B., Becker P., Krasovitsky L. (2013). Physical vapor deposition of peptide nanostructures. *Polymer Journal*.

[83] Rosenman G., Beker P., Koren I. (2011). Bioinspired peptide nanotubes: deposition technology, basic physics and nanotechnology applications. *Journal of Peptide Science*.

[84] Vasudev M. C., Koerner H., Singh K. M. (2014). Vertically aligned peptide nanostructures using plasma-enhanced chemical vapor deposition. *Biomacromolecules*.

[85] Yucel T., Cebe P., Kaplan D. L. (2011). Structural origins of silk piezoelectricity. *Advanced Functional Materials*.

[86] Iitaka Y. (1961). The crystal structure of *γ*-glycine. *Acta Crystallographica*.

[87] Ambujam K., Selvakumar S., Prem Anand D., Mohamed G., Sagayaraj P. (2006). Crystal growth, optical, mechanical and electrical properties of organic NLO material *γ*-glycine. *Crystal Research & Technology*.

[88] Srinivasan K. (2008). Crystal growth of *α* and *γ* glycine polymorphs and their polymorphic phase transformations. *Journal of Crystal Growth*.

[89] Selvarajan P., Glorium Arul Raj J., Perumal S. (2009). Characterization of pure and urea-doped *γ*-glycine single crystals grown by solution method. *Journal of Crystal Growth*.

[90] Zelenovskiy P., Vasileva D., Nuraeva A. (2016). Spin coating formation of self-assembled ferroelectric *β*-glycine films. *Ferroelectrics*.

[91] Heredia A., Bdikin I., Kopyl S. (2010). Temperature-driven phase transformation in self-assembled diphenylalanine peptide nanotubes. *Journal of Physics D: Applied Physics*.

[92] Bdikin I., Bystrov V., Delgadillo I. (2012). Polarization switching and patterning in self-assembled peptide tubular structures. *Journal of Applied Physics*.

[93] Vasilev S., Zelenovskiy P., Vasileva D., Nuraeva A., Shur V. Y., Kholkin A. L. (2016). Piezoelectric properties of diphenylalanine microtubes prepared from the solution. *Journal of Physics and Chemistry of Solids*.

[94] Gan Z., Wu X., Zhu X., Shen J. (2013). Light-induced ferroelectricity in bioinspired self-assembled diphenylalanine nanotubes/microtubes. *Angewandte Chemie International Edition*.

[95] Jenkins K., Kelly S., Nguyen V., Wu Y., Yang R. (2018). Piezoelectric diphenylalanine peptide for greatly improved flexible nanogenerators. *Nano Energy*.

[96] Nguyen V., Kelly S., Yang R. (2017). Piezoelectric peptide-based nanogenerator enhanced by single-electrode triboelectric nanogenerator. *APL Materials*.

[97] Lee J.-H., Heo K., Schulz-Schönhagen K. (2018). Diphenylalanine peptide nanotube energy harvesters. *ACS Nano*.

[98] Tao K., Xue B., Li Q. (2019). Stable and optoelectronic dipeptide assemblies for power harvesting. *Materials Today*.

[99] Ohnuki J., Sato T., Takano M. (2016). Piezoelectric allostery of protein. *Physical Review E*.

[100] Lemanov V., Popov S., Pankova G. (2003). Protein amino acid crystals: structure, symmetry, physical properties. *Ferroelectrics*.

[101] Denning D., Kilpatrick J. I., Fukada E. (2017). Piezoelectric tensor of collagen fibrils determined at the nanoscale. *ACS Biomaterials Science & Engineering*.

[102] Lee J. S., Yoon I., Kim J., Ihee H., Kim B., Park C. B. (2011). Self-assembly of semiconducting photoluminescent peptide nanowires in the vapor phase. *Angewandte Chemie International Edition*.

[103] Amdursky N. (2013). Enhanced solid-state electron transport via tryptophan containing peptide networks. *Physical Chemistry Chemical Physics*.

[104] Santhanamoorthi N., Kolandaivel P., Adler-Abramovich L. (2011). Diphenylalanine peptide nanotube: charge transport, band gap and its relevance to potential biomedical applications. *Advanced Materials Letters*.

[105] Tao K., Fan Z., Sun L. (2018). Quantum confined peptide assemblies with tunable visible to near-infrared spectral range. *Nature Communications*.

[106] Akdim B., Pachter R., Naik R. R. (2015). Self-assembled peptide nanotubes as electronic materials: an evaluation from first-principles calculations. *Applied Physics Letters*.

[107] Maia F. F., Freire V. N., Caetano E. W. S., Azevedo D. L., Sales F. A. M., Albuquerque E. L. (2011). Anhydrous crystals of DNA bases are wide gap semiconductors. *The Journal of Chemical Physics*.

[108] Tao K., Makam P., Aizen R., Gazit E. (2017). Self-assembling peptide semiconductors. *Science*.

[109] Andrade-Filho T., Ferreira F. F., Alves W. A., Rocha A. R. (2013). The effects of water molecules on the electronic and structural properties of peptide nanotubes. *Physical Chemistry Chemical Physics*.

[110] Kogikoski S., Sousa C. P., Liberato M. S. (2016). Multifunctional biosensors based on peptide–polyelectrolyte conjugates. *Physical Chemistry Chemical Physics*.

[111] Mei A., Luo X. (2019). The structural, electronic and optical properties of *γ*-glycine under pressure: a first principles study. *RSC Advances*.

[112] Yalcin S. E., O'brien J. P., Acharya A. (2019). *Conformation-induced conductivity switching in bacterial protein nanowires*.

[113] Rosenberg B. (1962). Electrical conductivity of proteins. *Nature*.

[114] Xu D., Watt G. D., Harb J. N., Davis R. C. (2005). Electrical conductivity of ferritin proteins by conductive AFM. *Nano Letters*.

[115] Yan H., Park S. H., Finkelstein G., Reif J. H., Labean T. H. (2003). DNA-templated self-assembly of protein arrays and highly conductive nanowires. *Science*.

[116] Amdursky N., Molotskii M., Aronov D., Adler-Abramovich L., Gazit E., Rosenman G. (2009). Blue luminescence based on quantum confinement at peptide nanotubes. *Nano Letters*.

[117] Yan X., Li J., Möhwald H. (2011). Self-assembly of hexagonal peptide microtubes and their optical waveguiding. *Advanced Materials*.

[118] Peng W., Cong G., Qu S., Wang Z. (2006). Synthesis and photoluminescence of ZnS:Cu nanoparticles. *Optical Materials*.

[119] Wang M., Xiong S., Wu X., Chu P. K. (2011). Effects of water molecules on photoluminescence from hierarchical peptide nanotubes and water probing capability. *Small*.

[120] Fan Z., Sun L., Huang Y., Wang Y., Zhang M. (2016). Bioinspired fluorescent dipeptide nanoparticles for targeted cancer cell imaging and real-time monitoring of drug release. *Nature Nanotechnology*.

[121] Li Y., Yan L., Liu K. (2016). Solvothermally mediated self-assembly of ultralong peptide nanobelts capable of optical waveguiding. *Small*.

[122] Handelman A., Apter B., Turko N., Rosenman G. (2016). Linear and nonlinear optical waveguiding in bio-inspired peptide nanotubes. *Acta Biomaterialia*.

[123] Sedman V. L., Adler-Abramovich L., Allen S., Gazit E., Tendler S. J. B. (2006). Direct observation of the release of phenylalanine from diphenylalanine nanotubes. *Journal of the American Chemical Society*.

[124] Amdursky N., Beker P., Koren I. (2011). Structural transition in peptide nanotubes. *Biomacromolecules*.

[125] Narayana Moolya B., Jayarama A., Sureshkumar M. R., Dharmaprakash S. M. (2005). Hydrogen bonded nonlinear optical *γ*-glycine: crystal growth and characterization. *Journal of Crystal Growth*.

[126] Kol N., Adler-Abramovich L., Barlam D., Shneck R. Z., Gazit E., Rousso I. (2005). Self-assembled peptide nanotubes are uniquely rigid bioinspired supramolecular structures. *Nano Letters*.

[127] Niu L., Chen X., Allen S., Tendler S. J. B. (2007). Using the bending beam model to estimate the elasticity of diphenylalanine nanotubes. *Langmuir*.

[128] Azuri I., Adler-Abramovich L., Gazit E., Hod O., Kronik L. (2014). Why are diphenylalanine-based peptide nanostructures so rigid? Insights from first principles calculations. *Journal of the American Chemical Society*.

[129] Azuri I., Meirzadeh E., Ehre D. (2015). Unusually large Young’s moduli of amino acid molecular crystals. *Angewandte Chemie International Edition*.

[130] Hartmann M. A., Weinkamer R., Zemb T., Fischer F. D., Fratzl P. (2006). Switching mechanics with chemistry: a model for the bending stiffness of amphiphilic bilayers with interacting headgroups in crystalline order. *Physical Review Letters*.

[131] Even N., Adler-Abramovich L., Buzhansky L., Dodiuk H., Gazit E. (2011). Improvement of the mechanical properties of epoxy by peptide nanotube fillers. *Small*.

[132] Yang L., van der Werf K. O., Fitié C. F. C., Bennink M. L., Dijkstra P. J., Feijen J. (2008). Mechanical properties of native and cross-linked type I collagen fibrils. *Biophysical Journal*.

